# Vogt–Koyanagi–Harada syndrome in the setting of COVID‐19 infection

**DOI:** 10.1002/ccr3.6617

**Published:** 2023-03-19

**Authors:** Tiffany Eatz, Jude Hassan Charles

**Affiliations:** ^1^ Department of Neurology, Miller School of Medicine The University of Miami Miami Florida USA

**Keywords:** comorbid, COVID‐19, infectious disease, rare, VKH

## Abstract

To report a case of Vogt–Koyanagi–Harada disease (VKH) in a 27‐year‐old male 2 weeks proceeding COVID‐19 infection onset. Severe complications of VKH can be avoided by early diagnosis and adequate treatment with corticosteroids and immunosuppressants. It is possible that COVID‐19 was a potential immunological trigger of VKH in our patient.

## Abstract 2

COVID‐19 can be a possible of Vogt–Koyanagi–Harada disease (VKH). Although rare, it is reasonable to consider VKH in COVID‐19 patients presenting with severe ocular symptoms since early treatment is key.

## 1 INTRODUCTION

Vogt–Koyanagi–Harada (VKH) disease is a T‐cell‐mediated multisystemic autoimmune inflammatory disorder characterized by skin, ocular, auditory, and neurologic involvement. The T‐cell‐mediated granulomatous intraocular inflammation is responsible for vitritis, disk edema, serous retinal detachments, and eventual sunset glow fundus.^1^ T cells target melanocytes, with an ensuing cascade leading to four distinct disease phases as follows: prodromal, uveitis, convalescent, and recurrent.^2^ VKH symptoms include headache, meningismus, hearing loss, poliosis, alopecia, and vitiligo.^3^ It is most prevalent in Asians, Native Americans, Hispanics, and Middle Easterners. If promptly and adequately treated, patients can experience good outcomes and avoid complications including sunset glow fundus, cataracts, glaucoma, subretinal fibrosis, and choroidal neovascularization. Some studies have reported the link between some viral infections such as cytomegalovirus (CMV) and Epstein–Barr virus (EBV) and the development of VKH. It has been postulated that similarity between peptides on melanocytes and some exogenous viral peptides lead to adverse T cells attacks on melanocytes‐containing tissues and lead to the symptoms seen in VKH.^4^ We report a case of a VKH syndrome in a patient diagnosed with COVID‐19.

## 2 CASE REPORT

A 27‐year‐old male patient with no significant past medical history presented with a two‐week history of intermittent unilateral headache, bilateral eye pain with photophobia, and phonophobia, followed by tinnitus and blurry vision, which ultimately progressed to total bilateral vision loss. His initial physical examination was unremarkable including vitals, except for total bilateral visual loss with preserved light perception. An ophthalmologic examination revealed normal intraocular pressure and panuveitis with serous retinal detachments bilaterally.

**FIGURE 1:**
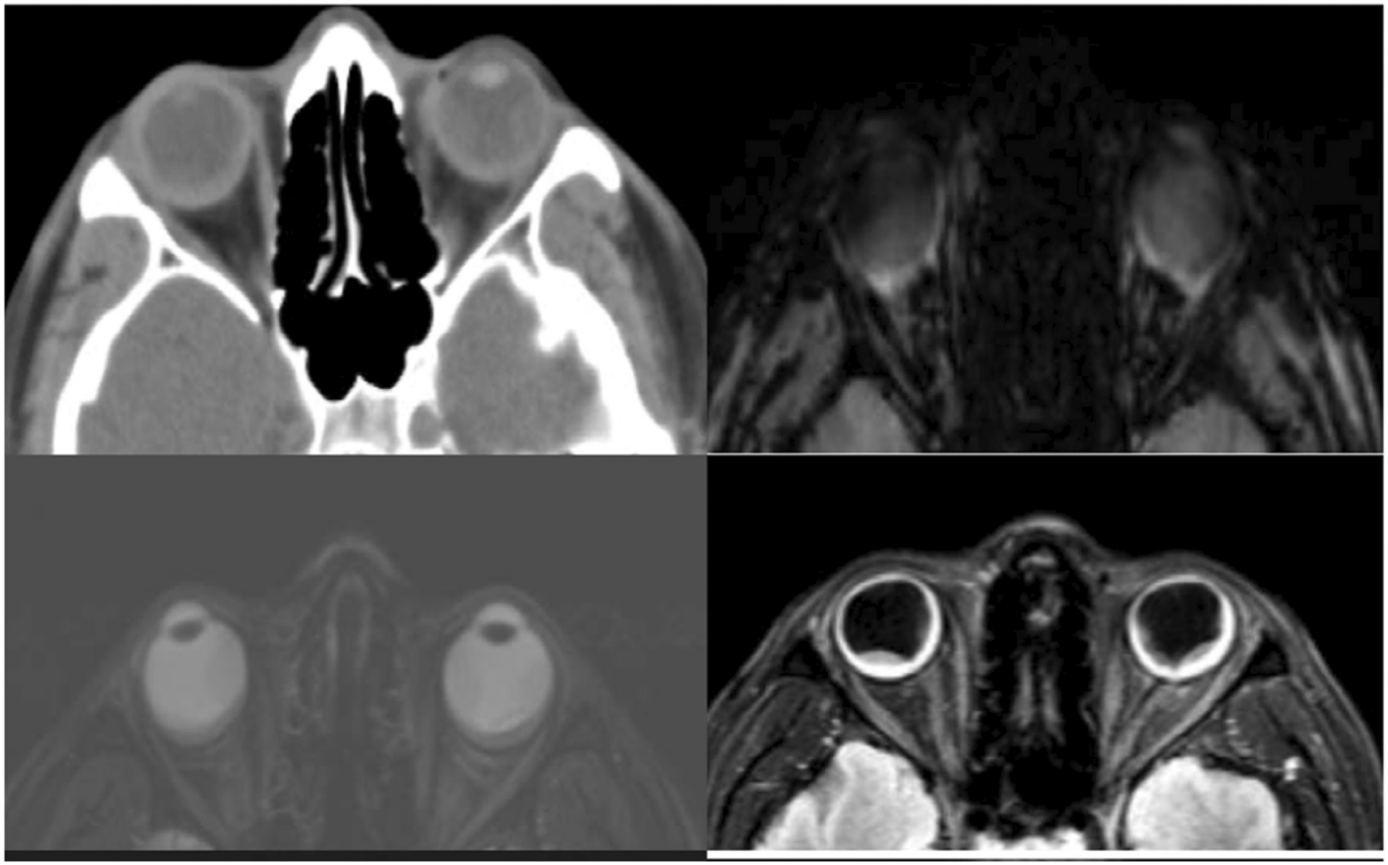
CT brain without contrast and sequential axial T1‐weighted MRI brain with and without gadolinium contrast demonstrating bilateral retinal detachment.

**FIGURE 2:**
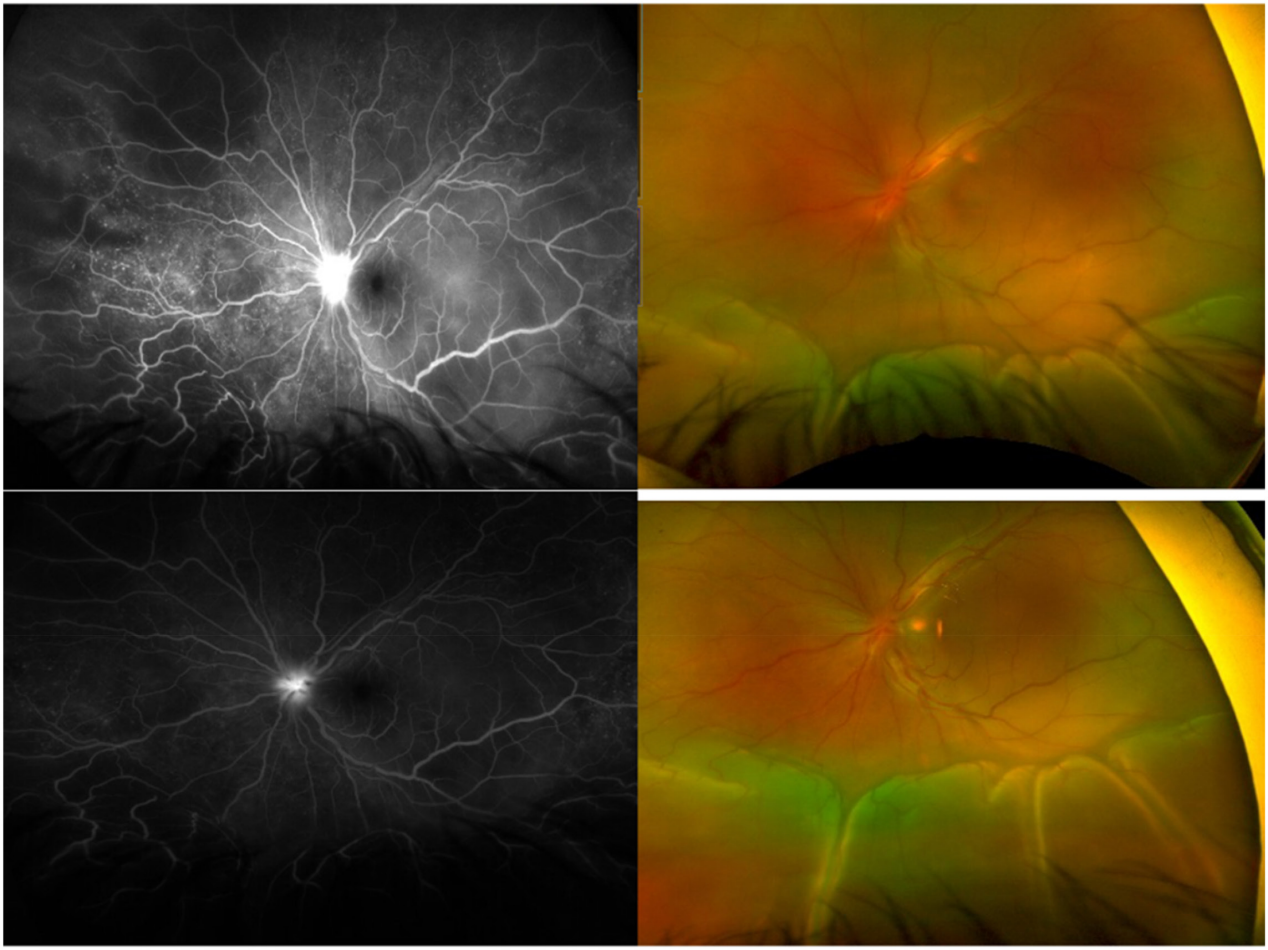
Fluorescein angiography bilateral eyes demonstrating fundus autofluorescence and mixed hyper and hypo fluorescence.

The patient was admitted for further evaluation. Infectious work‐up revealed positive COVID‐19 PCR. Other infectious and metabolic panels were unremarkable. Cerebrospinal fluid studies (CSF), venereal disease research laboratory test (VDRL), fluorescent treponemal antibody absorption test (FTA), QuantiFERON gold, flow cytometry, cytology, and cultures were all negative, except for moderate pleocytosis with lymphocytic predominance and elevated CSF protein. Cytology was also negative for malignancy. Computed tomography, B‐scan, and magnetic resonance imaging (MRI) showed bilateral retinal detachment with no optic nerve involvement (Figure 1). Fundus Fluorescein angiography (FFA)/indocyanine green (ICG) of the bilateral retinal vessels revealed mottled hyperfluorescence and hypofluerescence as well as fundus autofluorescence bilaterally associated with retinal pigment epithelium detachment consistent with VKH (Figure 2).

The patient was subsequently diagnosed with VKH. He was treated with high‐dose prednisone and was then discharged on a prednisone taper and methotrexate. Sixteen weeks later, the patient had a visual recovery to 20/60 on the left eye and 20/80 on the right eye, and his symptoms have since resolved.

## 3 DISCUSSION

Since VKH syndrome was firstly described, the need for diagnostic criteria became evident because of the complexity and the variety of symptoms associated with the disease. The American Uveitis Society (AUS) created the initial diagnostic criteria.^1^ However, its lack of specificity and sensitivity led to the development of the revised diagnostic criteria (RDC) for VKH disease created by Read et al. in 2001, which is the standard in current practice.^5^, ^6^ It includes five criteria: (1) the exclusion criteria for sympathetic ophthalmia, (2) the exclusion of other uveitis entities, (3) the ocular manifestations of the early and late stages, (4) the neurological/auditory findings, and (5) the integumentary findings.^7^ Based on the presence of all 5 criteria or less, VKH can be categorized as complete, incomplete, and probable.^1^ Some other attempts to optimize the criteria were made in China. Although their new criteria showed higher sensitivity compared to the RDC, external validity was limited, given their studies included only Chinese patients.^8^ Our patient presented with all the RDC criteria findings except for integumentary. However, integumentary findings are less common; therefore, our patient was diagnosed with VKH.

Some authors argue that the diagnosis of VKH can be established through clinical and imaging findings alone without the need for other ancillary tests, such as CSF analysis, and ultimately question their utility.^9^ However, when performed, CSF studies reveal a lymphocyte‐predominant pleocytosis in 75% of patients.^1^, ^10^ Our patient's CSF study also demonstrated this finding. It is important to note that CSF analysis contributes more to ruling out other infectious/inflammatory conditions than ruling in the diagnosis of VKH,^11^ especially because the differential diagnoses for VKH‐associated uveitis/meningitis are numerous.^12^ Those differentials include, but not limited to, other viral meningitis, uveitis, scleritis, syphilis, and carcinoma. Our patient's CSF, meningoencephalitis panel, syphilis testing, and cytology were negative, and thus, a diagnosis of VKH was favored.^13^

Imaging studies have been reported to be positive in 75%–100% of patients.^1^, ^5^, ^6^, ^12^, ^14^ In a series of 261 patients on optical coherence tomography, Yang et al.^8^ reported retinal detachment in 87% and hypo and hyperfluorescence on ICG and FFA in 91% of the patients. All of these findings were seen in our patient. Leptomeningeal enhancement is reported in the literature^15^ and was also observed in our patient.

There is no definitive etiological mechanism to explain the instigation of the pathogenesis of VKH. Over time, accumulating evidence has shown that genetic factors, including VKH disease‐specific risk factors (HLA‐DR4) and general risk factors for immune‐mediated diseases (IL‐23R), play a role in the development of VKH disease. However, dysfunction of the immunological response of both the innate and adaptive immune system, independent of congenital mutations, is involved as well, triggered by environmental and microbiological factors. These microbiological factors majorly consist of viruses, which have been discovered in patients with VKH disease. Symptoms of such viruses may even be observed in the VKH prodromal phase. Cellular and molecular mimicry is thought to be the triggering factor for VKH. For instance, tyrosinase enzyme is found mostly in melanocytes and has a similar peptide sequence as one of the proteins seen in CMV. Therefore, antibodies against CMV cross react with this enzyme and lead to VKH symptoms.^1^, ^4^ Patients with VKH had been reported to have positive CSF for EBV and CMV, though the pathogenesis is unclear.^2^, ^12^, ^16^

Although COVID‐19 PCR tests are positive for about 5–8 days after symptom onset, patients may continue to test positive for up to 3 months after their infection.^17^, ^18^ Therefore, it is possible that the immunological trigger for VKH in our patient was COVID‐19 if COVID infection onset was prior to the 2‐week history of VKH symptom onset, or during the prodromal VKH phase. Santamaria et al. reported one of multiple possible associations between COVID‐19 and VKH.^19^ Their patient presented 6 weeks after acute respiratory infection and was found to have positive COVID‐19 viral PCR in addition to typical VKH findings.^19^

Many other ocular manifestations of COVID‐19 infection have been reported in a systematic review by Aggarwal.^20^ He reported that ocular manifestations are found in 8%–15% of COVID‐19‐infected patients. Such manifestations include, but not limited to, follicular conjunctivitis, eye redness, ocular pain, and discharges.^20^ Panuveitis and unilateral optic neuritis were also reported.^21^

COVID‐19 has viral endovascular, neurological, and ophthalmological manifestations. The novel SARS‐CoV‐2 virus may cause atypical neurological impairment via ischemic and immunological alteration due to megakaryocyte infiltration.^22^ COVID‐19 has been demonstrated to cause not only both hemorrhagic and thrombotic stroke, venous sinus thrombosis, endotheliosis, but also dysfunction of smell and taste, muscle injury, Guillain–Barre syndrome, and encephalopathy, most likely mediated by neurotoxins and dysfunctional immunological response.^23^, ^24^, ^25^ Demonstrated by ophthalmological research, clinical observation, and analysis of social media posts,^26^ SARS‐CoV‐2 has also been found to instigate conjunctivitis, anterior uveitis, retinitis, and optic neuritis in patients.^27^, ^28^, ^29^ It is not beyond reason that given COVID‐19's immunological, neurological, and ophthalmological manifestations, it was the possible inducing microbiological trigger in the development of VKH disease in our patient.

Although the definitive underlying mechanism by which COVID‐19 can trigger VKH has yet to be elucidated, decreased immune tolerance, increased antigenicity, spike protein toxicity, and molecular mimicry are plausible. VKH has even been reported in patients after COVID‐19 vaccines in various instances.^30^, ^31^ Nevertheless, we also acknowledge that due to a high rate of COVID‐19 infection, the association between COVID‐19 and VKH could be fortuitous.

## 4 CONCLUSIONS

Vogt–Koyanagi–Harada is a rare and potentially debilitating disorder in which severe complications can be avoided by early diagnosis and adequate treatment. It is imperative to keep up with advancing radiological and technological advancements to enable optimization of the rate of VKH diagnosis. Corticosteroids and immunosuppressants remain the mainstay of treatment for VKH. COVID‐19 could be a potential immunological trigger of VKH in our patient if COVID‐19 infection onset was prior to the 2‐week history of VKH symptom onset or during VKH prodromal phase. Further investigation is necessary to establish whether this hypothesis is valid.

## AUTHOR CONTRIBUTIONS

All authors attest that they meet the current ICMJE criteria for Authorship.

## CONFLICT OF INTEREST

The following authors have no financial disclosures or personal conflicts of interest: JC and TAE.

## CONSENT

Written informed consent was obtained from the patient to publish this report in accordance with the journal's patient consent policy. Consent to publish this deidentified case report was obtained.

## Data Availability

The data that support the findings of this study are openly available at https://pubmed.ncbi.nlm.nih.gov and https://www.scopus.com/home.uri.
